# Long-term outcomes and health-related quality of life 20 years after pediatric liver transplantation

**DOI:** 10.1007/s13304-023-01608-2

**Published:** 2023-08-03

**Authors:** Davide Cussa, Angelica Pino, Silvia Catalano, Chiara Montini, Federico Assanti, Licia Peruzzi, Michele Pinon, Pier Luigi Calvo, Marco Spada, Damiano Patrono, Fabrizio Gennari, Jean-Bernard Otte, Mauro Salizzoni, Renato Romagnoli

**Affiliations:** 1 General Surgery 2U-Liver Transplant Unit, Azienda Ospedaliero Universitaria Città della Salute e della Scienza di Torino, University of Turin, Ospedale Molinette, Corso Bramante 88-90, 10126 Turin, Italy; 2 Pediatric Nephrology Unit, Department of Pediatrics, Azienda Ospedaliero Universitaria Città della Salute e della Scienza di Torino, Ospedale Infantile Regina Margherita, Turin, Italy; 3 Pediatric Gastroenterology Unit, Department of Pediatrics, Azienda Ospedaliero Universitaria Città della Salute e della Scienza di Torino, Ospedale Infantile Regina Margherita, Turin, Italy; 4 Department of Pediatrics, University of Turin, Azienda Ospedaliero Universitaria Città della Salute e della Scienza di Torino, Ospedale Infantile Regina Margherita, Turin, Italy; 5 Pediatric Surgery Unit, Department of Pediatrics, Azienda Ospedaliero Universitaria Città della Salute e della Scienza di Torino, Ospedale Infantile Regina Margherita, Turin, Italy; 6 Abdominal Transplantation Unit-Service de Chirurgie Digestive et Transplantation, Cliniques Universitaires Saint-Luc, Brussels, Belgium

**Keywords:** Liver transplantation, Pediatric, Health-related quality of life, Long-term outcomes, Immunosuppression

## Abstract

Pediatric liver transplantation is a challenging surgical procedure requiring complex post-transplant patient management. Liver transplantation in children should ensure long-term survival and good health-related quality of life (HR-QOL), but data in the literature are conflicting. With the aim of investigating survival and psychosocial outcomes of patients transplanted during childhood, we identified 40 patients with ≥ 20-year follow-up after liver transplantation regularly followed up at our Institution. Clinical charts were reviewed to retrieve patients’ data. Psychosocial aspects and HR-QOL were investigated by an in-person or telephonic interview and by administering the WHOQOL-BREF questionnaire through an online form. Ten- and 20-year patient survival was 97.5% (95% CI 92.8–100%), whereas 10- and 20-year graft survival was 77.5% (65.6–91.6%) and 74.8% (62.5–89.6%), respectively. At last follow-up visit, 31 patients (77.5%) were receiving a tacrolimus-based immunosuppression. Twelve (32.4%) patients obtained a university diploma or higher, whereas 19 (51.4%) successfully completed high school. 81.1% of patients were active workers or in education, 17.5% had children, and 35% regularly practiced sport. 25 patients answered to the WHOQOL-BREF questionnaire. More than 60% of respondents did not report any disability and the perceived physical status was invariably good or very good. Median scores for physical health, psychological health, social relationships, and environment were 16.6, 14.7, 16, and 15, respectively. Pediatric liver transplantation is associated with excellent long-term survival and good HR-QOL. Psychological health and environment represent areas in which support would be needed to further improve HR-QOL.

**Supplementary Information:**

The online version contains supplementary material available at 10.1007/s13304-023-01608-2.

## Introduction

As compared to adult liver transplantation (LT), pediatric LT is characterized by unique features concerning indications, surgical technique, and post-LT management. Given the constant improvement of survival outcomes after pediatric LT over the last decades [1–14], the focus has shifted toward management and prevention of long-term complications, including renal dysfunction, impaired linear growth, post-transplant lymphoproliferative disease, and other side effects of chronic immunosuppression [15, 16]. Adherence to immunosuppression therapy and health-related quality of life (HR-QOL) represent crucial aspects during childhood and adolescence, with some studies having suggested inferior HR-QOL in pediatric recipients of an LT [5, 17, 18]. Furthermore, the recent COVID-19 pandemic may have exacerbated preexisting difficulties in coping with the constraints associated with being an LT recipient [19, 20].

Data concerning very long-term outcomes (≥ 20 years of follow-up) after pediatric LT are limited [10, 21, 22] and lack an in-deep evaluation of HR-QOL. Furthermore, other aspects like scholarity, work occupation, sport practicing, and parental status have been largely overlooked.

The ultimate goal of pediatric LT would be allowing long-term survival, good HR-QOL, and full reintegration as a functioning member of the society. Thus, with the aim of providing a comprehensive picture of long-term outcomes after pediatric liver transplantation, we conducted a retrospective study on pediatric LT recipients (age < 18 years) with a minimum follow-up of 20 years, with a particular focus on social integration aspects and HR-QOL.

## Patients and methods

The pediatric liver transplant program at our Institution was started in 1995 and 186 pediatric LTs have been performed since. Furthermore, Italian patients from our region transplanted at the Abdominal Transplantation Unit—Cliniques Universitaires Saint-Luc in Brussels, Belgium are regularly followed up at our outpatient clinic, in the setting of a more than 3 decade-long collaboration between our two Institutions. After discharge from hospital and until 3-month follow-up, patients have blood tests checked every 15 days and a clinic appointment with an US scan monthly. After 3 months, follow-up schedule includes monthly blood tests and a clinic appointment, including a US scan, every 6 months, unless otherwise clinically indicated. After 5 years, clinic appointments and US scans are scheduled yearly. Protocol liver biopsies are performed 1, 3, and 5 years after LT, and every 5 years thereafter. Fibroscans are performed yearly except in the years in which a liver biopsy is programmed. Importantly, patients and their families have the possibility, in case of any concern, to directly contact by phone a member of the team, which is available every day during working hours. From our Institutional database, we identified 40 patients transplanted before January 1st, 2004, i.e., with a minimum follow-up of 20 years by the end of 2023. Data concerning the indication for LT, surgical technique, immunosuppression, as well as patient and graft survival were retrieved from clinical charts.

To investigate aspects concerning social integration, an in-person or telephonic interview was submitted to all included patients. Patients were asked whether they were in education or actively working, regularly practicing sport, and whether they were married and/or had children. Additional information was collected about the highest scholarly degree, the kind of work, the type and of frequency of sport activity, whether they were living alone or with a family, and the number of children.

Health-related QOL was investigated by administering the World Health Organization QOL-BREF (WHOQOL-BREF) questionnaire, which is a validated tool for the assessment of HR-QOL (https://www.who.int/tools/whoqol). Briefly, WHOQOL-BREF is a 26-question questionnaire based on the WHOQOL-100. It allows assessing HR-QOL relative to four domains, namely physical health, psychological, social relationships, and environment. Importantly, the questionnaire includes some preliminary questions for stratification purposes. The WHOQOL-BREF Italian translation available on the WHO website (https://www.who.int/tools/whoqol/whoqol-bref/docs/default-source/publishing-policies/whoqol-bref/italian-whoqol-bref) was used to set up an online survey using the Google Forms app. According to WHO instruction, answers to each question were scored on a scale from 1 (worst score) to 5 (best score). Answers to questions 3, 4, and 26 (negatively phrased items) were recoded to match the scores obtained from other items. The mean of the scores for each domain question was then multiplied by 4, resulting in a scale from 4 (worst score) to 20 (best score) for each domain. The study was conducted according to the principles of the Istanbul and Helsinki declarations.

Unless otherwise specified, data are presented as counts (percentage) and median (interquartile range). Survival analysis was conducted using the Kaplan–Meier method and survival curves compared using the log-rank test. Data analysis and visualization was performed using R version 4.2.3 (R: A language and environment for statistical computing. R Foundation for Statistical Computing, Vienna, Austria. URL https://www.R-project.org/).

## Results

Some 40 patients with 20-year follow-up transplanted between January 30th, 1996 and November 11th, 2003 were included in the study. Of these, 17 were transplanted at our Institution, whereas 23 were transplanted at the Abdominal Transplantation Unit—Cliniques Universitaires Saint-Luc. The 17 patients from our center represented 9.1% of pediatric liver transplants performed at our Institution, whereas the 23 from Cliniques Saint-Luc represented 76.7% of pediatric liver transplant performed at other Institutions that are followed up at our center. One patient died during follow-up, whereas two were lost to follow-up. All other patients responded to the in-person or telephonic interview, whereas 25 (67.6%) answered also to the online WHOQOL-BREF questionnaire.

Baseline patient characteristics and outcomes are summarized in Table 1. Median age at LT was 1.4 years, with 29 (72.5%) of recipients being male. Biliary atresia was by far the most frequent indication for LT in 24 (60%) of cases. Nineteen (47.5%) patients were transplanted with a whole graft from a deceased donor, 16 (40%) with a left lateral segments graft from a deceased donor, and 5 (12.5%) received a left lateral sector from a living donor. Three patients underwent combined liver–kidney transplantation, with grafts procured in all cases from deceased donors. Ten (25%) patients underwent retransplantation and 2 (5%) were retransplanted twice.

**Table 1:** Baseline patient characteristics and long-term outcomes

*n*	40
Gender	
F	11 (27.5)
M	29 (72.5)
Age	1.4 [0.8, 3.3]
Indication for LT	
Biliary atresia	24 (60.0)
Liver cirrhosis–fibrosis	5 (12.5)
Malignancy	4 (10.0)
Alagille syndrome	3 (7.5)
Inborn error of metabolism	2 (5.0)
Primary hyperoxaluria type I	1 (2.5)
Wilson	1 (2.5)
Type of graft	
Deceased donor—whole	19 (47.5)
Deceased donor—LLS	16 (40.0)
Living donor—LLS	5 (12.5)
Combined liver–kidney transplant	3 (7.5)
Re-LT	10 (25.0)
Number of transplants	
1	30 (75.0)
2	8 (20.0)
3	2 (5.0)
Maintenance IS	
BID Tac	7 (18.4)
BID Tac + MMF	2 (5.3)
BID Tac + prednisone	2 (5.3)
CyA	3 (7.9)
MMF	3 (7.9)
None	1 (2.6)
OD Tac	15 (39.5)
OD Tac + MMF	4 (10.5)
OD Tac + prednisone	1 (2.6)
Scholarity	
University diploma or higher	12 (32.4)
High school	19 (51.4)
Middle school	2 (5.4)
None	4 (10.8)
Working or in education	30 (81.1)
Had children	7 (17.5)
Practice sport	14 (35.0)

Data are expressed as number (counts) or median (interquartile range)

*LLS* left lateral sector, *LT* liver transplantation, *BID* bis in die, *Tac* tacrolimus, *MMF* mycophenolate mofetil, *CyA* cyclosporin A, *OD* once daily

**Fig. 1:**
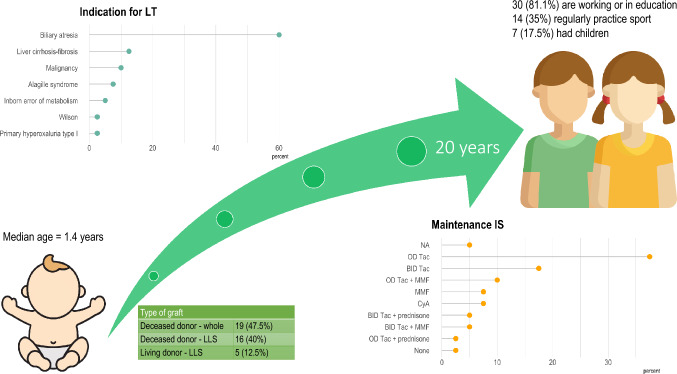
A visual summary of main study findings. Icons downloaded from Freepik.com

At last follow-up visit, most patients (*n* = 31; 77.5%) were receiving a tacrolimus-based immunosuppression (once daily, *n* = 20; twice daily, *n* = 11) (Fig. 1). Three (7.9%) were on cyclosporin A or mycophenolate mofetil monotherapy, respectively. Only one patient was off immunosuppression as a result of his decision to stop taking immunosuppressant, which did not result in acute rejection. As this was discovered months after he had stopped medications, immunosuppressant therapy was not resumed.

**Fig. 2:**
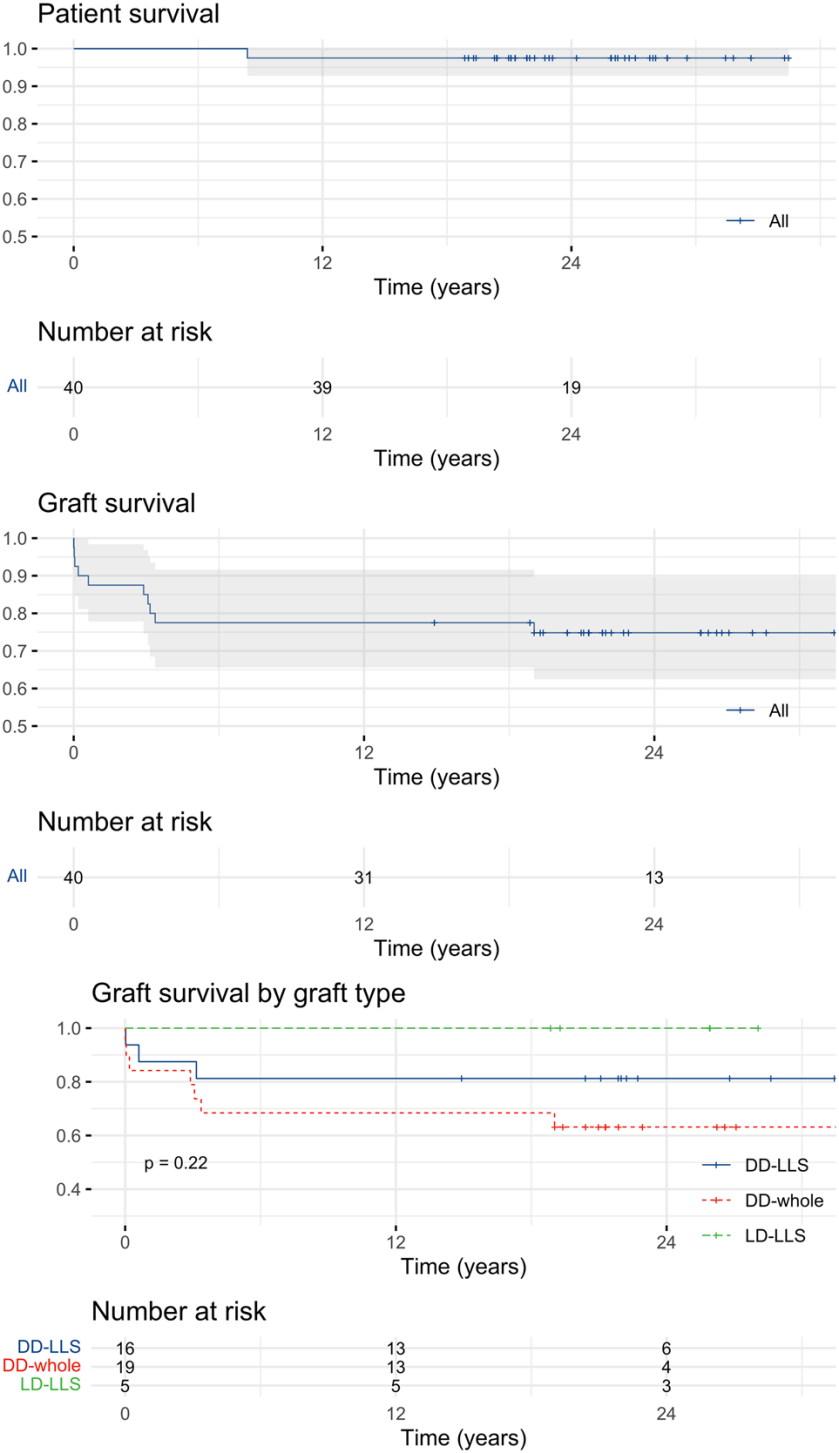
Kaplan–Meier survival curves depicting patient and graft survival in the whole cohort. Third panel represents graft survival according to the type of graft (*p* value from log-rank test)

Patient and graft survival are depicted in Fig. 2. Median follow-up was 23 (21–27.8) years. Patient survival at 10 and 20 years was 97.5% (95% confidence interval 92.8–100%), whereas 10- and 20-year graft survival was 77.5% (65.6–91.6%) and 74.8% (62.5–89.6%), respectively. The only patient death in this series was due to post-transplant lymphoproliferative disease. Causes of graft loss differed according to its timing. Among the four patients who lost their graft within 90 days from LT, hepatic artery thrombosis was observed in 3 (75%) patients, whereas primary non-function in 1 (25%). Seven grafts were lost later than 90 days after LT due to chronic rejection (*n* = 4, 57.1%), de-novo autoimmune hepatitis (*n* = 1, 14.3%), death with functioning graft (*n* = 1, 14.3%), and undetermined cause (*n* = 1, 14.3%). Median patient and graft survival were not reached during follow-up. Recipients of a whole graft from a deceased donor showed inferior graft survival as compared to the other groups (Fig. 2, third panel). This difference, however, did not reach statistical significance.

Results of the in-person or telephonic interview are summarized in Table 1. Twelve (32.4%) patients obtained a university diploma or higher, whereas 19 (51.4%) successfully completed high school. The majority of patients (*n* = 30, 81.1%) were active workers or in education, 7 (17.5%) had children, and 14 (35%) regularly practiced sport.

**Fig. 3:**
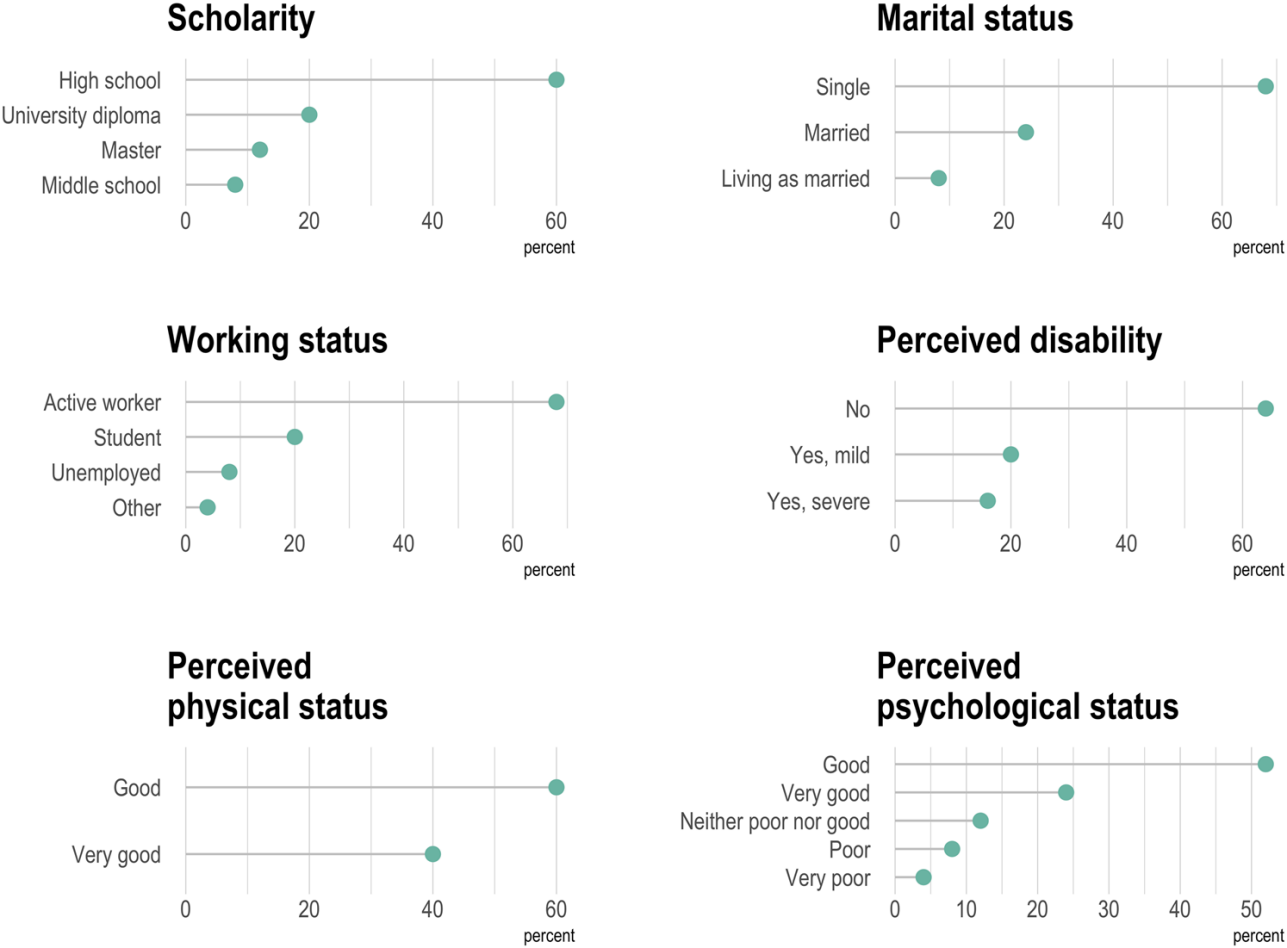
Line plots with dots (lollipop plots) depicting answers to the introductory questions of the WHOQOL-BREF questionnaire by the 25 participants who answered to the survey

**Fig. 4:**
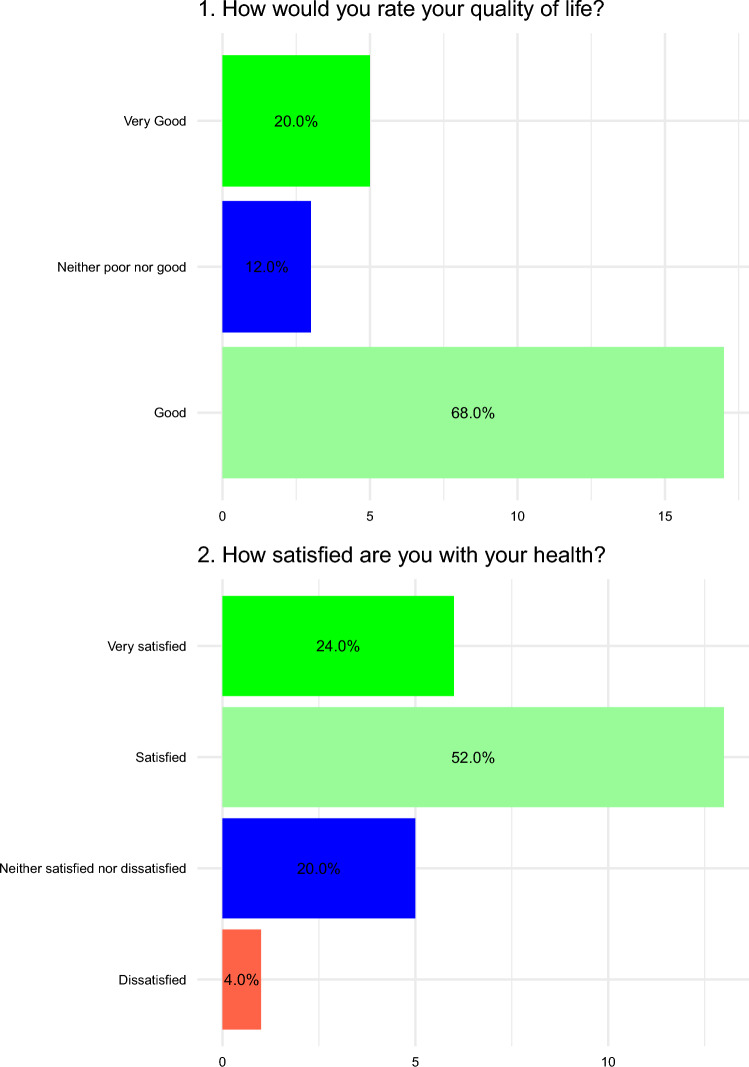
Bar plots with percentages representing answers to the first two questions of the WHOQOL-BREF questionnaire

Figure 3 depicts answers to the introductory WHOQOL-BREF questions from the 25 participants who took the survey, substantially confirming what already shown by the interview. Notably, more than 60% of respondents did not report perceiving any disability and the perceived physical status was good or very good in 60% and 40% of cases, respectively. Results concerning psychological status were somewhat less brilliant, with 8% and 4% perceiving a poor or very poor psychological status, respectively. Figure 4 depicts answers to the first two questions of the WHOQOL-BREF questionnaire, which are not included in any domain calculation. Overall, 68% and 20% of respondents would rate their quality of life as good or very good, respectively, whereas 52% and 24% were satisfied or very satisfied with their health. Answers to remaining questions are reported as supplementary material (Supplementary Figs. 1–4). Overall, median scores for each domain were 16.6 for physical health, 14.7 for psychological health, 16 for social relationships, and 15 for environment (Table 2).

**Table 2:** Summary statistics of the scores relative to the four domains of WHOQUOL-BREF questionnaire

	Min	Q1	Median	Mean	Q3	Max
Physical	11.4	15.4	16.6	16.6	18.3	20.0
Psychological	8.7	13.3	14.7	14.4	15.3	19.3
Social relationships	5.3	13.3	16.0	14.3	16.0	17.3
Environment	8.5	13.5	15.0	14.7	16.0	17.5

## Discussion

Our results show excellent long-term patient survival after pediatric LT and suggest that, despite the constraints linked to the status of LT recipient, social integration and good HR-QOL are frequent. It is noteworthy that more than 80% of patients in these series were actively working or in education at the moment they were interviewed, and that the perceived physical status was invariably good or very good.

As defined by World Health Organization, HR-QOL is “an individuals’ perception of own position in life in the context of the culture and value systems in which they live and in relation to own goals, expectations, standards and concerns” [23]. Liver transplantation in the pediatric age has the disruptive capacity of reversing the fate of young patients affected by incurable liver diseases or metabolic defects. It would be tempting to focus exclusively on excellent survival outcomes while overlooking the necessity to allow them to grow and develop as functioning members of the society. Long-term survival as chronically ill patients would be, however, at least partially deceiving. According to our data, pediatric LT is not only associated with excellent long-term patient survival but also with a good HR-QOL in most cases, with the majority of patients living a life very much close to what could be defined as normal.

In general, our results are in line with those from the literature [24–26]. In 2002, Atkinson et al. [3] showed that most pediatric LT recipient experiences normal growth and development, with about 90% of the patients transplanted in their childhood presenting with intellectual development appropriate for their age. In 2015, the Birmingham group explored the struggles and difficulties of young LT recipients in an article with a significant title: “‘It’s hard but you’ve just gotta get on with it’—The experiences of growing-up with a liver transplant” [18]. In this paper, 13 patients transplanted during their childhood or adolescence were interviewed, revealing how the perception of being different from their peers was frequent. This was mainly caused by the perception of their scars, the need to take immunosuppressants, and limitations to some everyday activities, like practicing contact sports. Konidis et al. [17] highlighted how engagement in full time work or study is associated with enhanced physical health. Mayer et al. [27] investigated long-term psychosocial outcomes in pediatric patients transplanted at Hannover Medical School before 2002. Most patients transitioned without problems to adult age and showed a high degree of self-esteem and social integration.

Our data also point to areas where there appears to be some room for improvement. Despite being in general quite satisfied by their physical health, patients were less so concerning psychological health and environment, suggesting that better support would be needed in these domains to further improve HR-QOL. Adolescence and young adulthood are ages in life in which social relationships are of utmost importance. The limitations inherent to the status of LT recipient may lead to the perception of be tagged as “different” and social isolation. While this did not appear to be a dramatic issue in our series, psychological support for patients and their families would be of undoubtful value. Also, while 35% percent of patients regularly practicing sport may seem encouraging, this should be taken as a starting point for further improvement, also given the well-known benefits of regular physical activity in solid organ transplant recipients [28]. Participating to associations and groups promoting sport practice among LT recipient has been demonstrated to improve physical fitness and health, and would have the added value of stimulating social interactions [29, 30]. Almost all patients were still on immunosuppressants 20 or more years after LT. How hard is going to be the management of the expectable side effects of immunosuppression during the subsequent follow-up remains an open question. Efforts aimed at minimizing or weaning immunosuppressive therapy have been mostly unsuccessful at our Institution (unpublished data) and it is concerning to observe how the landscape of immunosuppressive medications has not changed in the last two decades. Finally, in our series, a quarter of patients required a second graft, with chronic rejection being the main cause of late graft loss despite protocol biopsies were regularly performed [10]. This highlights the need for better and less-invasive tools to monitor the efficacy of immunosuppressive therapy, like donor-derived cell-free DNA [31], and for a further expansion of pediatric donor pool by implementing liver splitting [32], utilizing novel dynamic approaches to organ preservation [33, 34] and exploiting DCD donors as a valuable source of pediatric liver grafts [2, 13].

Limitations of our study include study design and its retrospective, single-center nature, and its relatively small sample size, which limit the representativeness and generalizability of the findings. In particular, the lack of a matched comparison group of non-transplanted patients did not allow to put into context the good outcomes observed in our cohort. Response rate to the WHOQOL-BREF questionnaire was 25/37 (67.6%) and, although this figure is in line with those from the literature, a source of bias may originate from the fact that patients with a better perceived quality of life could have been more prone to answer the survey. Strengths are represented by the granularity of data, which was possible thanks to the decades-long relationship with our patients, and by the utilization of a validated HR-QOL assessment tool that was administered by an online form, a modality with which most patients were at ease with.

## Conclusions

Pediatric liver transplantation is nowadays associated with excellent long-term survival outcomes and good HR-QOL. Psychological health and environment are areas where interventions would be required to further improve HR-QOL. Larger, multicenter, prospective studies are necessary to provide more robust evidence and to ascertain whether these good results will persist in the longer term follow-up.

## Supplementary Information

Below is the link to the electronic supplementary material.

Supplementary file1 (DOCX 67 KB)

## Data Availability

The data that support the findings of this study are available on request from the corresponding author. The data are not publicly available due to privacy restrictions.
