# Supplementary material for: Long-term outcomes and health-related quality of life 20 years after pediatric liver transplantation
Source: Updates Surg. 2023 Aug 3;75(6):1549–57. doi: 10.1007/s13304-023-01608-2 (PMC10435421; doi:10.1007/s13304-023-01608-2)
Supplement: Supplementary file 1 — Supplementary file1 (DOCX 67 KB) [file 13304_2023_1608_MOESM1_ESM.docx]

**Long-term outcomes and quality of life 20 years after pediatric liver transplantation**

**Supplementary material**

The following plots depict answers to questions 3 to 26 of the WHOQOL-BREF questionnaire from 25 participants to the study, grouped by domain.

Colour code linked to the score for each question are as follows:

| **Colour** | **Score** |
| --- | --- |
| Magenta | 1 (worst) |
| Brown-red | 2 |
| Blue | 3 |
| Light green | 4 |
| Green | 5 (best) |
